# Evaluation of a Piezo-Actuated Sensor for Monitoring Elastic Variations of Its Support with Impedance-Based Measurements

**DOI:** 10.3390/s19010184

**Published:** 2019-01-07

**Authors:** Hector A. Tinoco, Carlos I. Cardona, Fabio M. Peña, Juan P. Gomez, Samuel I. Roldan-Restrepo, Maria A. Velasco-Mejia, Daniel R. Barco

**Affiliations:** 1 Computational and Experimental Mechanics Laboratory, Universidad Autónoma de Manizales, Antigua Estación del Ferrocarril, edificio fundadores, C.P. 170001 Manizales-Caldas, Colombia; ivancg19@gmail.com (C.I.C.); fabiope@autonoma.edu.co (F.M.P.); danielbarco6@gmail.com (D.R.B.); 2 Institute of Physics of Materials, Czech Academy of Sciences, Zizkova 22, 61662 Brno, Czech Republic; 3 Central European Institute of Technology (CEITEC)/Brno University of Technology, Technická 10, 61600 Brno, Czech Republic; 4 Department of Oral Health, Universidad Autónoma de Manizales, Antigua Estación del Ferrocarril, Edificio Fundadores, C.P. 170001 Manizales-Caldas, Colombia; jgomez@autonoma.edu.co (J.P.G.); mariaandreavelascomejia@hotmail.com (M.A.V.-M.); 5 Centro de Innovación Roldán, C.P. 050024 Medellín-Antioquia, Colombia; sirr1965@gmail.com

**Keywords:** structural health monitoring, bio-monitoring, piezo-actuated sensor, electromechanical impedance technique, elastic variations, vibration through teeth

## Abstract

This study exposes the assessment of a piezo-actuated sensor for monitoring elastic variations (change in Young’s modulus) of a host structure in which it is attached. The host structure is monitored through a coupling interface connected to the piezo-actuated device. Two coupling interfaces were considered (an aluminum cone and a human tooth) for the experimental tests. Three different materials (aluminum, bronze and steel) were prepared to emulate the elastic changes in the support, keeping the geometry as a fixed parameter. The piezo device was characterized from velocity frequency response functions in pursuance to understand how vibration modes stimulate the electrical resistance through electrical resonance peaks of the sensor. An impedance-based analysis (1–20 kHz) was performed to correlate elastic variations with indexes based on root mean square deviation (RMSD) for two observation windows (9.3 to 9.7 kHz and 11.1 to 11.5 kHz). Results show that imposed elastic variations were detected and quantified with the electrical resistance measurements. Moreover, it was demonstrated that the sensitivity of the device was influenced by the type of coupling interface since the cone was more sensitive than the tooth in both observation windows. As a final consideration, results suggest that bio-structures (fruits and bone, among others) could be studied since these can modify naturally its elastic properties.

## 1. Introduction

In the structural health monitoring (SHM) field, predictive and preventive strategies are playing an important role in the prognosis of catastrophic scenarios in order to guarantee safety and reliability during the service of the structures. Currently, different monitoring applications have been implemented with piezoelectric technologies due to its implementation easiness and relatively low cost in comparison with other nondestructive inspection techniques. The structural integrity analysis based on changes of dynamic characteristics requires a permanent monitoring to detect, locate and quantify damages by means of in-situ diagnosis techniques, such as have been implemented in aircraft wings, space shuttles, concrete, turbine rotor blades, and pressure vessels, among others [1,2,3,4,5]. In general, SHM presents advantages with respect to the preventive traditional way of diagnosis since it minimizes the operative costs of maintenance because it eliminates unnecessary technical checks. Moreover, with this technology the structures acquire an intelligence component since these could provide information of its functionality for which were designed.

SHM technologies are being extrapolated to biological fields with the purpose of detecting specific changes in bio-system properties. Bio-structures modify its intrinsic conditions by natural effects or by modifications in the biological equilibrium. Some examples of bio-systems that can modify its properties are fruits [6], bone tissue [7], and plants [8], among others. These changes are detected traditionally by several techniques that involve the use of chemical components that identify any variation in a passive way in laboratory tests. It indicates that there is no inspection and monitoring in-situ by the challenges that exist with this type of procedures. From a physical point of view, geometric and elastics variations in bio-structures could be considered as entities with invariant properties (slow changes) and therefore traditional SHM techniques could be applied in the same way as in conventional structures.

Electromechanical impedance technique (EMI) permits to detect structural changes in high frequency (typically >20 kHz) by means of a self-diagnosis of piezo-transducers [5,9,10,11]. Mechanical impedance variations (damage or structural variations) of the host structure are determined through a correlation obtained by electrical impedance measurements in piezo-transducers (PZT). The quantification of any change is performed through indices computed between a healthy structural condition and a monitoring state. Some applications have shown the feasibility of the technique in applications of bio-monitoring. For instance, [12] using PZT for the assessment and detection of different types of damage in a chicken’s femur, in which damages were identified though the EMI technique. In the study carried out by [13], piezoelectric patches were used in human and rabbit bones as biomedical sensors. EMI evaluated the feasibility of the technique when bone was modified by different controlled conditions as crack existence, fractures and density variations. In other applications for bone, [14] presented an experimental study to detect a simulated healing process by applying the EMI technique. The applied methodology consisted in putting a healing agent in particular cracks in an isolated bone to be monitored by a PZT-needle sensor. Results showed that the computed index increased with the time of healing bone until it reached a stable value. It indicated that the method was capable of detecting that the bone cracks were fully recovered. The electrical impedance measurements were taken using an AD5933 evaluation board which is low-cost; this aspect is pointed out since the majority of nondestructive techniques require expensive equipments. Assessment of human bone conditions is a significant challenge toward future diagnosis of physiological state in-situ. Bone tissue is not only responsible for providing an adaptive structural support of the body, but also makes available a reservoir for the complex metabolism of calcium, showing its biomedical importance [15].

A study reported by [16] applied the EMI technique with piezo-sensors in a non-bonded configuration to an artificial arm (bones covered with skin and tissue, silicone-based coating). The overall results demonstrated good prospects since the conductance signals correlated well in all experiments in the healthy and damaged conditions. [17] connected a PZT to an intact pig limb with soft tissue (muscle, fat, skin, etc.) obtained from a butcher. Simulated fractures in the bone were measured by electromechanical impedance spectroscopy and dynamic stimuli were sent to a receiver located externally on the skin surface. The presented methodology was able to accurately detect cuts in the bone as shallow as 2 mm and distinguish between different depths of cuts in the bone.

Other approaches of bio-monitoring have been explored in the dentistry area. For instance, dental implant stability requires a permanent evaluation of the prosthesis since the load-bearing depends on the integrations of bone–implant system. For this purpose, [18] assessed a monitoring system over a controlled experiment. Dental implants were entrenched in two in vitro bone types, namely an extremity and a costal bovine bone. The inverse of the osseointegration process (bone degradation) was simulated by immersing the samples into a solution of nitric acid for several hours. Results showed that variations in the calcium percentage were quantified through the conductance measurements. This work was extended by [19] from a numerical approach including an experimental validation performed by [20] in another study. They suggested a hypothesis based on the EMI technique which consisted in bonding a PZT-5A4E (Piezo System, Inc., Cambridge, MA, USA) to an abutment screwed into two bovine bone samples to measure the electrical admittance. This had the aim to monitor and assess the degree of healing of the peri-implant tissue by means of the electrical impedance of PZT. The experiments were conducted by using a dental cement paste Pulp Canal Sealer EWT (PCS) (SybronEndo, Glendora, CA, USA) to interlock the implant to in vitro bovine bone samples, which simulated the osseointegration process. The experimental results obtained in eight implant sites in the same bone evidenced similar trends in the indexes calculated. The root mean square (RMS) index rapidly decreased within the first few hours and then stabilized during the last 6 h of monitoring. These results inspired different work done by [21,22,23] to use teeth embedded in different substrates with the objective to detect structural variations. In those studies, the teeth were used as coupling interfaces to monitor the changes in the substrate which allowed to show that it is feasible to diagnose the bone as a future application. The substrates tried to mimic the bone portion that contains the teeth. The consideration of a mechanical coupling interface between the sensor and the host structure have been described in other studies as [24,25]. However, in the mentioned works (teeth in substrates), the electro-dynamic behavior of the piezo-device and coupling interface effects on the electrical signatures have not been studied yet, which is the main contribution of this study.

This paper presents an electro-dynamic assessment of a piezo-actuated sensor for monitoring the elastic variations (change in Young’s modulus) of a substrate (host structure) with fixed geometric conditions and variable Young’s modulus. The host structure acts as a main support of the coupling interface (aluminum cone or human tooth) that joints the piezo device with it. The substrate was made of three different materials (aluminum, bronze and steel) to simulate the elastic changes in the monitoring process with the EMI technique.

## 2. Materials and Methods

### 2.1. Electromechanical Impedance Technique (EMI) Principles

Piezoelectric materials present electro-mechanical properties that have been exploited in structures with different purposes, for instance to monitor the structural integrity in the frequency spectrum [9,25,26]. If a PZT is deformed by dynamic external forces, electrical charges are moved through the poling direction producing an electric field through thickness (sensor case). In the opposite case, when an electrical field is applied on it, it changes its shape (actuator case). This effect permits it to deform a host structure for obtaining indirect information through electrical measurements [27,28,29]. Two approaches can be applied; the first one is to measure the electrical signatures caused by local deformations (direct sensor) and the second one is associated with the measurement of electrical properties in the PZT (indirect sensor), i.e., electrical impedance. In recent years, the second approach has demonstrated great potential due to the capacity of correlating structural variations (physical changes) with the electrical impedance of the material [29,30,31,32].

According to [28], a PZT is considered an electrical element that can generate two states when combined with other electrical components (resistance, capacitor and inductor), i.e., open circuit and short circuit. Electrically, a PZT is a resistive-capacitive element. It means that there is no inductance since it has the capacity to store energy by short time periods. Therefore, the rules applied to electrical circuits are analogously valid for PZTs. In a PZT, the electrical impedance is described in general terms as

(1) $$Z_{P}^{E}(\omega )=\frac{V(\omega )}{i(\omega )}=R(\omega )+X(\omega )j$$

where ω is the frequency, R(ω) is the electrical resistance and X(ω) is the reactance which can be inductive or capacitive; being V(ω) and i(ω) the input voltage and output current. In practical terms, the constants inside X(ω) can be experimentally determined using a parameter identification from the electrical impedance if those parameters are unknown. It is important to denote that the electrical resistance is a constant value as a pure electrical component, but it depends on the frequency in a PZT [33].

**Figure 1:**
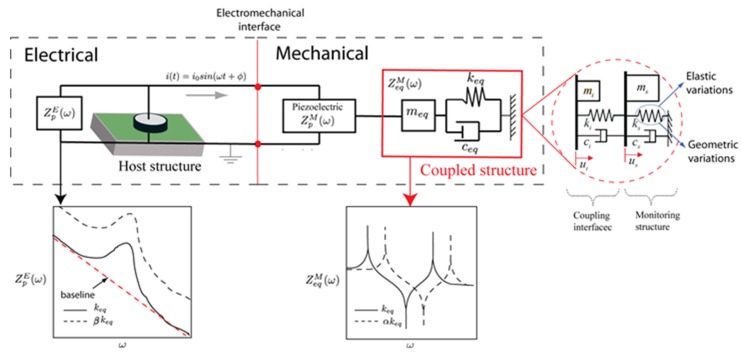
Scheme of a coupled electromechanical system of one degree of freedom with stiffness variations in the host structure.

Figure 1 shows a simple scheme that couples a mechanical system with an electrical system [34] determined electromechanical admittance $Y_{P}^{E}(\omega )$ for a one-dimensional model of a PZT bonded to a structure as follows:

(2) $$Y_{P}^{E}(\omega )=\frac{1}{Z_{P}^{E}(\omega )}=\frac{2\omega jw_{p}l_{p}}{h_{p}}\left[e_{33}\left(1-j\delta \right)-d_{31}^{2}{\bar{y}}^{E}+\left(\frac{Z_{p}^{M}(\omega )}{Z_{p}^{M}(\omega )+Z_{eq}^{M}(\omega )}\right)d_{31}^{2}{\bar{y}}^{E}\left(\frac{\tan(kl_{p})}{kl_{p}}\right)\right]$$

where $h_{p}$, $l_{p}$ and $w_{p}$ are the thickness, the length and the width of the piezoelectric material. $d_{31}$ is the piezoelectric strain coefficient corresponding to x(1)−z(3) coordinates, ${\bar{y}}^{E}=y^{E}(1+\eta )$ is the complex Young’s modulus of the piezoelectric sheet at constant electric field and $e_{33}$ is the electric permittivity. η and δ denote both mechanical loss and dielectric loss factors. $Z_{p}^{M}(\omega )$ and $Z_{eq}^{M}(\omega )$ are mechanical impedances of the PZT and structure. Equation (2) shows that the mechanical impedances of both structures are related with the electrical impedance. The application of this principle is called EMI. In Figure 1, a scheme is observed that mechanical impedances are coupled by the dynamic interaction between $Z_{p}^{M}(\omega )$ and $Z_{eq}^{M}(\omega )$ (equivalent impedance). However, in this study, $Z_{eq}^{M}(\omega )$ is represented by a system of two degrees of freedom composed by a monitoring structure and a coupling interface. Usually, a coupling interface serves as a probe of the piezo-device for monitoring the host structure as detailed in the work done by [25,26]. In a mechanical system with two degrees of freedom, the mechanical parameters (masa, stiffness and damping) are related in the following way with the equivalent impedance as follows

(3) $$Z_{eq}^{E}(\omega )=\frac{2\omega \left(-\omega^{2}m_{i}+j\omega c_{i}+k_{i}\right)\left(-\omega^{2}m_{s}+j\omega c_{i}+k_{i}+k_{s}\right)-{\left(j\omega c_{i}+k_{i}\right)}^{2}}{j\omega \left(-\omega^{2}m_{s}+j\omega c_{i}+k_{i}+k_{s}\right)}$$

where the subscripts “*i*” and “*s*” refer to coupling interface and monitoring structure. As electromechanical impedance theory suggests, each modification in the stiffness $k_{eq}$ (coupled structure) is reflected on the electrical impedance, indicating that the stiffness can be modified by $\alpha k_{eq}$, where α is a function that depends on the frequency. The stiffness changes can be produced by geometric and elastic variations. Geometric changes can be induced by damages meanwhile elastic variations are related with degradation in structures or intrinsic changes in its properties such as biosystems. With the EMI technique, changes can be quantified by means of indexes that are computed typically by comparing two electrical impedance signals. The first one is a signal established when the structure is considered healthy and this is called baseline. The second one is the monitoring signal. The quantitative comparison is carried out by calculating different indices such as root mean square deviation (RMSD), correlation coefficient deviation metric (CCDM), mean absolute percentage deviation (MAPD) and others reported in the literature [32,35,36,37]. In this study, RMSD index will be used to quantify the changes in the electromechanical impedance. RMSD index is based on the Euclidean norm and it is the most widely used in the EMI technique; it is defined in our study by

(4) $$RMSD=\sqrt{\frac{\sum_{i=1}^{n}{\left(R_{i(M)}-R_{i(\mathrm{Ref})}\right)}^{2}}{\sum_{i=1}^{n}{\left(R_{i(\mathrm{Ref})}\right)}^{2}}}\times 100$$

where $R_{i(M)}$ is the electrical resistance of the monitoring signal, $R_{i(\mathrm{Ref})}$ corresponds to the values of the resistance of a signal taken as reference or baseline. This is defined from a linear trend of the analyzed signal as described in Figure 1.

### 2.2. Working Principles of the Piezo-Actuated Sensor

**Figure 2:**
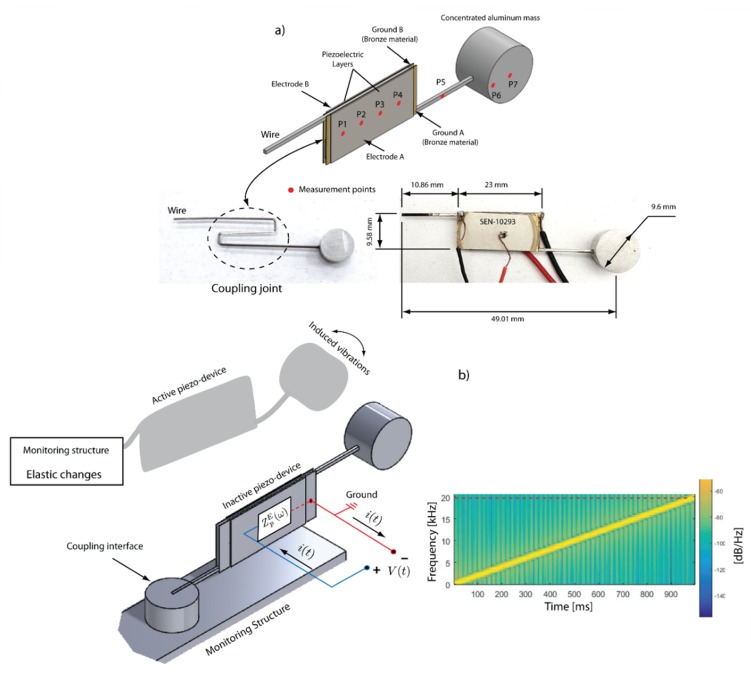
(**a**) Piezo-device design, dimensions and measurement points. (**b**) Piezo-device working principle.

This section presents and develops the description and design of an electromechanical piezo-actuated sensor, as illustrated in Figure 2a. The device is proposed to evaluate the elastic variations of a support or substrate in which a mechanical coupling element is allocated. The support is called monitoring structure or host structure, as detailed in Figure 2b. The following elements compose the electromechanical device: a flexible wire made of stainless-steel with the shape of “S” (coupling joint) is the principal element. The wire has the dimensions of 0.021 × 0.025 in, commonly used in orthodontic treatments. Two piezoelectric patches are bonded to the coupling joint to arrange a composite joint. A supplementary cylindric aluminum mass (1.27 g) is embedded to the end of the wire. PZTs used in the sensor device are SEN10293 ROHS (SparkFun Electronics, Niwot, CO, USA) and these were cut into rectangular shapes in order to cover the coupling joint; the piezo patches are the same used in the study [29,38]. Piezo-device design, dimensions and configuration are illustrated in Figure 2.

The piezo-actuated sensor is activated by applying a harmonic voltage (linear sweep) in such way that oscillatory deformations are produced, a scheme is described in Figure 2b. Therefore, bending is induced in the wire due to a force system generated by the PZTs. The physical purpose of the piezo sensor is to monitor the host structure through the coupling interface. This is an element that helps to transfer the vibrations between the piezo-actuated sensor and the monitoring structure. It can have any geometry, for this study a tooth and a piece of aluminum will be used as coupling interfaces. Piezo-actuated devices present advantages when these are used in monitoring tasks, since the whole dynamic system depends on three parameters that are invariants; mass, stiffness and damping. If these any of these parameters are modified, changes can be observed in the electrical impedance [36,39]. All experiments carried out in this study were performed with an unique device which is shown in Figure 2a.

### 2.3. Velocity Measurements of the Piezo-Actuated Sensor

For the assessment and comprehension of the piezo-device dynamic behavior, velocity measurements are taken when it is clamped in a mechanical press. It means that the coupling interface and the monitoring structure are not considered in the initial analysis. The main objective is to identify some mechanical resonances of the piezo-device itself to compare it with the resonance peaks of the electrical impedance.

**Figure 3:**
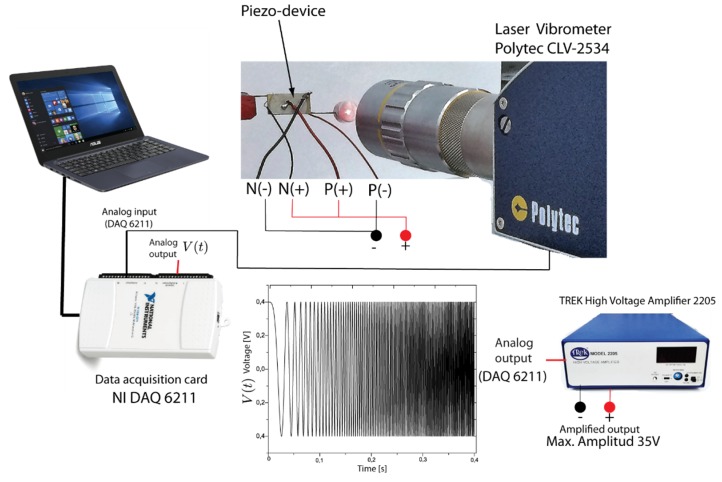
Experimental setup for the velocity measurements.

The experimental setup is defined by a computer, a data acquisition card (NI DAQ 6211) which has 16 analog inputs (16 bit, 250 kS/s), a power amplifier TREK high voltage amplifier 2205 (TREK Inc., Lockport, NY, USA) and a POLYTEC CLV-2534 (Polytec Inc., Auburn, MA, USA) laser vibrometer. A scheme of the experimental setup is shown in Figure 3. The piezo-device is fixed in one end of the wire to a mechanical press for the measurements. The red color marked points (Figure 2a) are established as measurement points of velocity which are distributed for the piezo-patches (P1 to P4), mass (P6 to P7) and wire (P5). Piezoelectric transducers are connected to the voltage output of the power amplifier in which a broadband chirp excitation signal of 35 V is applied in the input connector. The frequency of the input signal sweeps from 0 kHz to 20 kHz over a 1 s window. A sample frequency was set in 100 K samples/s in the data acquisition system. Simultaneously, the laser vibrometer was focused on the marked points in the piezo device to register the velocity by means of a laser beam that should be focused to avoid scattered data. For the experiment, seven tests were performed in order to obtain different measurements on the piezo-device in each detailed measurement point.

### 2.4. Experimental Setup for Structural Monitoring with the EMI Technique

**Figure 4:**
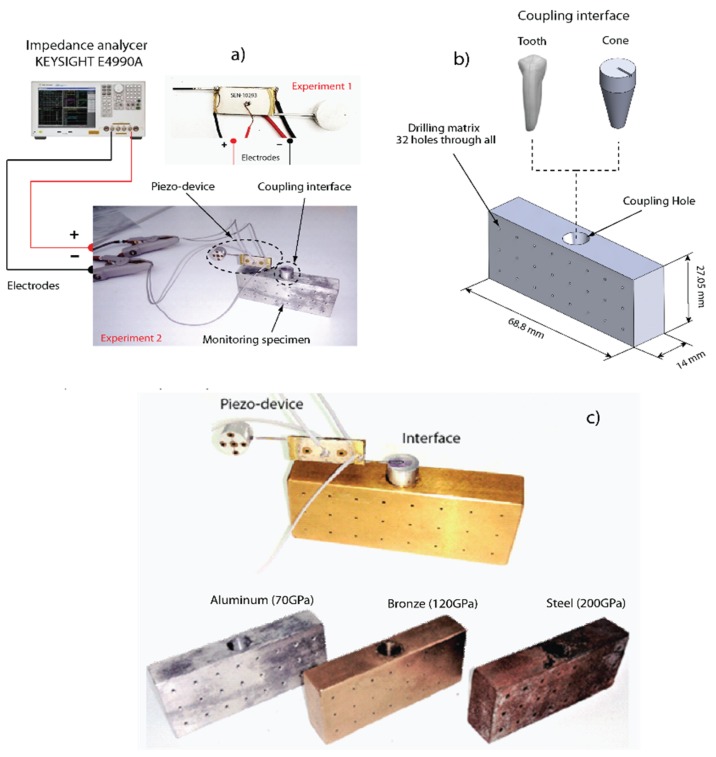
(**a**) Experimental setup for electromechanical impedance measurements. (**b**) Dimensions and materials for the monitoring structure including the coupling interface. (**c**) Materials for the experiment.

An experimental setup was settled in order to correlate the mechanical resonances of the piezo-actuated sensor from electrical impedance applying the EMI technique. The identification will permit to choose which resonances belong to mechanical responses that can be monitored from the electrical measurements by EMI, as explained in Section 2. The electrical impedance was measured using an impedance analyzer (E4990A, Agilent, Palo Alto, CA, USA) in two proposed experiments; in the first one, the piezo-actuated sensor is isolated, and in the second one it is coupled to a monitoring structure as detailed in Figure 4a.

The activation principle of the piezo device consists of exciting both piezoelectric patches simultaneously applying a harmonic voltage and reading the current response signals that pass through it using the impedance analyzer [40]. The sweep setup of the signal was defined from 1 to 20 kHz with 400 points of resolution in the selected bandwidth.

For the searched application, the piezo-device was coupled to three different metal specimens (aluminum, bronze and steel) to emulate a variation of the elastic modulus in the monitoring structure as shown in Figure 4b. Dimensions of the specimens are illustrated in the figure, as well as both coupling interfaces (human tooth and conic element). Each specimen has the same dimensions and the experiment was organized in the following way: the piezo-device is bonded into a slot of the coupling interface which in turn is joined to the monitoring structure. In order to guarantee stability conditions, the monitoring structure was fixed to a rigid structure in the bottom surface during the test. Epoxy adhesive (adhesive Loctite 4981) was used to bond all parts. To clarify the use of the human tooth, it is considered that biological structures such as bone modify their mechanical properties depending on different factors such as stress application, natural remodeling and metabolic diseases; the experiment pretends to show that the piezo device can identify the elastic variations independently of the coupling interface used.

Six tests were performed in the chosen materials (Aluminum, Bronze, Steel) using both coupling interfaces. Elastic properties of each material were provided according to the material supplier as 70 GPa (Aluminum), 120 GPa (Bronze), and 200 GPa (Steel). For the first one, three tests were done with the aluminum conic interface (Cone) coupled to the three specimens; and in the second one, another three tests were carried out with a premolar tooth coupled to each specimen. Coupling interfaces were bonded with epoxy adhesive Loctite 4981. It is also important to highlight that all three specimens have the same dimensions and geometrical configuration in order to focus our study on stiffness changes due exclusively to elastic changes done in the monitoring structure.

## 3. Results and Discussion

### 3.1. Velocity Measurements in the Piezo-Device

**Figure 5:**
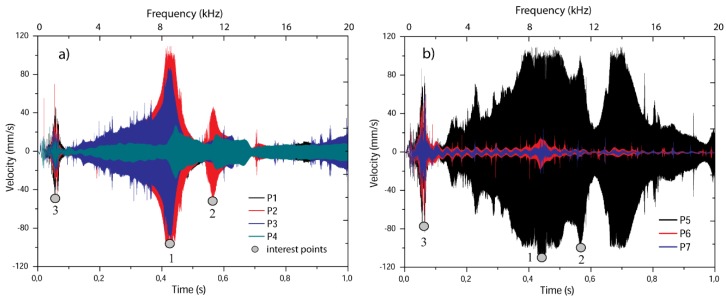
Velocity measurements. (**a**) Piezoelectric patch (P1, P2, P3, P4). (**b**) Wire and mass (P5, P6, P7).

Figure 5 consolidates all velocity signals measured with the vibrometer laser (POLYTEC CLV 2534) for the seven points marked in the piezo-device as described in Figure 2; four in the PT (P1 to P4), two in the mass (P6 and P7) and one in the wire (P5). The excitation signal was applied on each piezo patch as a harmonic chirp in an inverse way (inverse electric field for each PZT) to induce bending in the device. It means that the mass displacements were amplified. The connection scheme of the experiment is detailed in Section 2.3.

Velocity signals measured in one piezoelectric patch are illustrated in Figure 5a. For obtaining the measurements, the laser was focused in a normal direction to the electrodes of the piezo patch. There were observed different velocity magnitudes in the time domain; it indicates that the device takes different vibration shapes in specific values of the time-frequency spectrum established for a window of 1 s and 0–20 kHz. Vibration shapes refer to the specific deformation (geometric profile) that represent the kinematics of the device in any frequency value. It is analyzed that there are three velocity maximum transitions (grey points) that reflect the resonant nature of the device. The higher amplitude is observed close to 0.42 s, in which the points P2 and P3 take the maximum values (central points), but P1 and P4 achieve about 20% of the maximum velocities. For the same time, Figure 5b shows that P5 (on the wire) presents the same amplitude as P2 and P3 in 0.42 s, approximately. However, the velocity in the mass reaches 10% of it, revealing that the velocities are much lower than the other points. It denotes that the mass presents small motions and the body of the device is bending. The second largest velocity amplitude is observed in 0.57 s for both Figure 5a,b. In this peak, half of the piezo patch is deformed which bends the wire to its higher amplitude. A third peak is identified in 0.005 s, in which the mass and the wire present greater velocities compared with those of the piezo patches.

**Figure 6:**
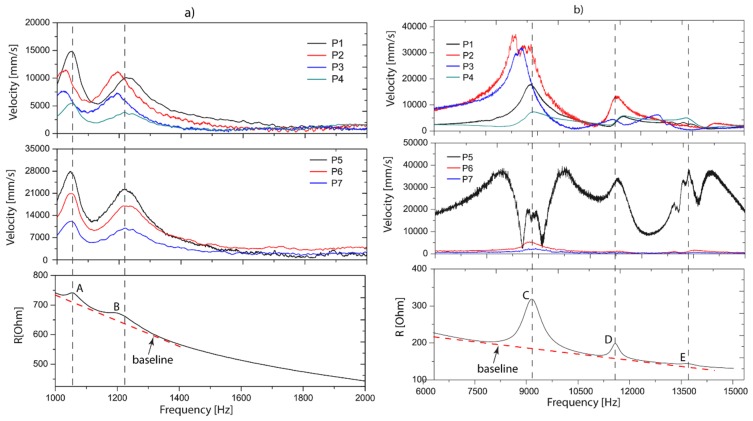
Comparison between velocity and electrical resistance, (**a**) 1–2 kHz. (**b**) 6–15 kHz.

To determine the frequency values that corresponds to these maximum velocities, Fourier’s transform was computed and plotted in Figure 6. All obtained frequency response velocity functions are particularly detailed in the following section. All velocities in frequency are compared with the electrical impedance results.

### 3.2. Comparison between Velocity and Electrical Impedance Measurements

This section deals with comparisons of measurements obtained for the velocity and electrical resistance in the frequency domain (real part of the electrical impedance). Electrical resistance measurements were also carried out with the piezo-device mounted in a mechanical press (isolated) as the velocity experiments. Electrical resistance was measured using the impedance analyzer (E4990A, Agilent, Palo Alto, CA, USA) shown in Figure 4a (experiment 1).

Figure 6 compares the velocities and electrical resistance in two groups with the aim to show the correlation between the mechanical and electrical peaks in the following frequency ranges of 1–2 kHz (Figure 6a) and 6–15 kHz (Figure 6b). These frequency intervals were defined by an exploratory data analysis which implied to cut the interval 2–6 kHz. Dashed black lines represent the values in frequency that give rise to peaks.

Two peaks are visible in the range of 1 to 2 kHz, with the first peak at 1046 Hz (A) and a second peak at 1220 Hz (B). It is observed that the electrical resistance presents two smoothed peaks in the same frequency values, approximately. This correlation indicates that reflected peaks in the electrical resistance correspond with mechanical resonances induced by the dynamics of the device. Nevertheless, that influence is low since the relative amplitude (height from the baseline) of the peak is low, indicating that the piezoelectric patches are being deformed slightly by the vibrations. This can be verified reconstructing the vibration shape with the amplitudes of these peaks. In other words, for monitoring purposes the vibration modes should be favorable to deform the PZTs since it will reflect a higher amplitude in the electrical resonances. Methods based on the idea of electromechanical impedance and vibration modes require a dynamic analysis to obtain a major sensitivity in the electrical signatures when the monitoring structure is modified. For this reason, this interval (1–2 kHz) is not considered as a monitoring spectrum.

In the range of 6–15 kHz, three peaks were identified in the electrical resistance, with the first peak at 9.1 kHz (C), second peak at 11.5 kHz (D) and a third peak at 13.7 kHz (E) as shown in Figure 6b. As in the first case (Figure 6a), as for the second one (Figure 6b), it is evident that the electrical peaks have a coincidence with the kinematic ones. Moreover, it is seen that relative amplitude for peak A is 130 Ohms and for B 37 Ohms, approximately. Comparing the relation between the relative amplitudes of Peak C and D in the velocities, D represents 30.5% of the amplitude of C. In the electrical resistances, this relation is 28.5%. According to the above, the results demonstrated that electrical resonances strictly depend on the vibration mode in the resonant frequencies.

The correlation of mechanical parameters through the electrical impedance shows a big advantage in SHM applications. It is not necessary to measure kinematic parameters in localized degrees of freedom to verify the resonances of the whole structure. It implies that a PZT is able to capture the mechanical resonances if these influence the deformation of it, as observed in Figure 6b, first one figure, peak C (velocity graph), whereas P1 and P4 have 1/3 and 1/5 of the amplitudes of P2 and P3. It means that the piezo patch is deformed by bending. To evaluate the piezo-actuated sensor in monitoring conditions, experiment 2 was proposed, as described in Figure 4a which is discussed in the next section.

### 3.3. Assessment of Young’s Modulus Variation in the Monitoring Structure

The main idea of the proposed piezo device is to track changes in the electrical resistance when the elastic properties undergo variations in monitoring structure. As Figure 4 details, the experiment emulates different elastic conditions with three specimens of different materials. We want to remark that the elastic variations in-situ can occur in some bio-structures such as bone [41,42] and fruits [43], among others [44,45,46].

**Figure 7:**
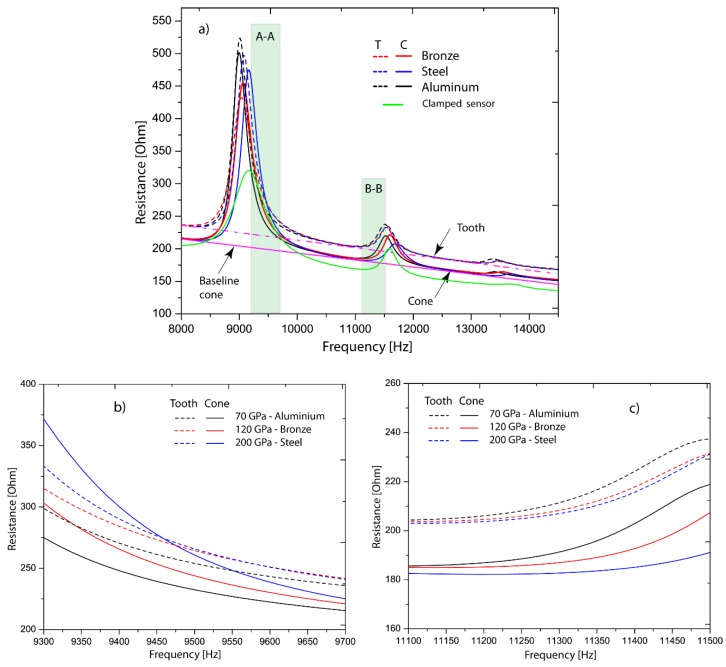
(**a**) Electrical resistance for experiments 1 and 2 in the frequency spectrum 8 kHz–15 kHz. (**b**) Electrical resistance signals in the first monitoring window (9.3–9.7 kHz). (**c**) Electrical resistance signals in the second monitoring window (11.1–11.5 kHz).

Figure 7a shows six curves that represent the electrical resistances of all the experimental tests composed by three measurements with a human tooth and three with an aluminum cone. All signals were acquired in the bandwidth 8–15 kHz according to the analysis described in the previous section. The experiments were designed considering the following working hypothesis, which states that independent of the coupling interface (cone and tooth), it should generate similar results in both experiments.

As seen in Figure 7a, the piezo device presents a structure of three peaks supported by a baseline that is demarked in pink color. The baseline serves as reference pattern of each coupling interface. It is noted that the baseline is moved upwards when the device is connected to the coupling interfaces, which in turn are embedded to the metallic specimens. It means that the principal conditions of the signal (three peaks and linear trend) are kept in other levels of electrical resistance and these are modified only by the coupling interface. As discussed in Section 3.1 and Section 3.2, the piezo device is activated in vibration modes that deform it.

Other changes occurred in the relative amplitudes (height from its baseline) of the peaks that were incremented, especially the first one. This is caused by the clamped conditions (piezo device) since the vibration mode is less constrained (lower stiffness in the boundary). Also, the peaks were slightly shifted with respect to the peaks of the green curve (clamped device). The presence of new elements in the piezo device caused changes in its mechanical resonances as naturally expected, because these are directly affected by the mass/stiffness relation of the whole system. However, as results showed, it can be inferred that the mechanical resonances did not vary dramatically keeping the mechanical impedance of the sensor as the principal structure of the signal.

Analyzing the curves, there were found two observability windows where the signals were sensitive to the change of elastic variations. The searching parameter was based on the progressive changes of the signal when Young’s modulus was increased. The first window was found in the range of 9.3–9.7 kHz, which is located at the right side of the first peak and labeled as A-A, and the second monitoring window is established in the interval of 11.1–11.5 kHz at the left side of the second peak, labeled as B-B, as illustrated in Figure 7b,c.

In Figure 7b, the electrical resistance shifts upwards when Young’s modulus increases its value. This happens because the resonance (peak one) is moving to the right since the whole system gains some stiffness from 70 GPa to 200 GPa. In Figure 7c, the contrary is observed since curves moved downwards, but the main effect is the same as peak one. Analyzing in detail the results, it is seen that peak two shifts to the right also. The differences in both observation intervals are focused on the location of the window since one was taken after the resonance and the other one before it.

**Figure 8:**
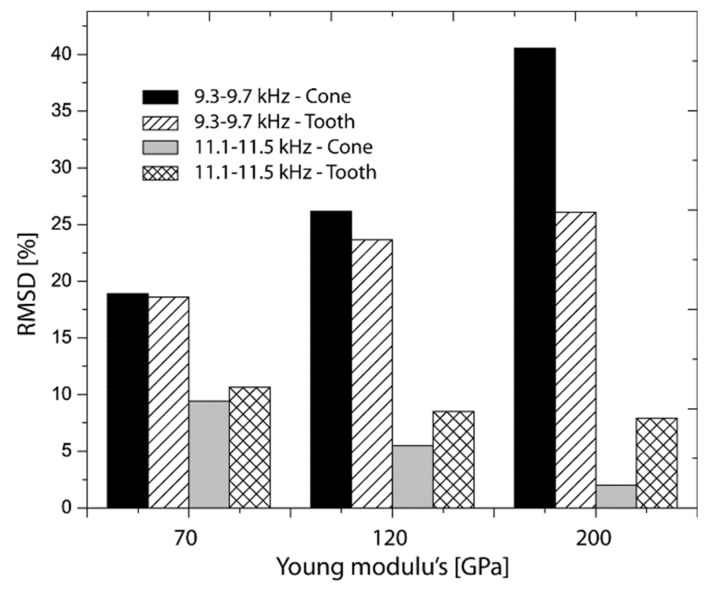
Root mean square deviation (RMSD) calculated for electrical resistances obtained in A-A and B-B.

In order to quantify and monitor elastic variations using the observation windows, RMSD index was calculated (see Equation (4)) and the results are summarized in Figure 8. It is important to point out that, to determine the RMSD index, it was necessary to set a linear reference (baseline) for each coupling interface as illustrated Figure 7a. In both Figure 8a,b, there are compared the apparent relations between Young’s modulus and the RMSD index for each coupling interface. Figure 8a shows that RMSD indexes increased with the variation of Young’s modulus in both cases. In Figure 8b, indexes decreased as expected since the electrical resistance relation were inverse to those determined in Figure 7a. In both cases, RMSD of the cone is more sensitive to the elastic changes between 12–40% and 17–2%, contrasted with 7.5–25% and 16–8% for the tooth. In conclusion, it is observed that in both cases it was possible to quantify the elastic variations from the frequency ranges established for each coupling interface. For bio applications, it important to denote that bio-structures can modify its elastic capacity significantly in lower increments and therefore the exposed methodology could be applied in higher frequency intervals.

**Figure 9:**
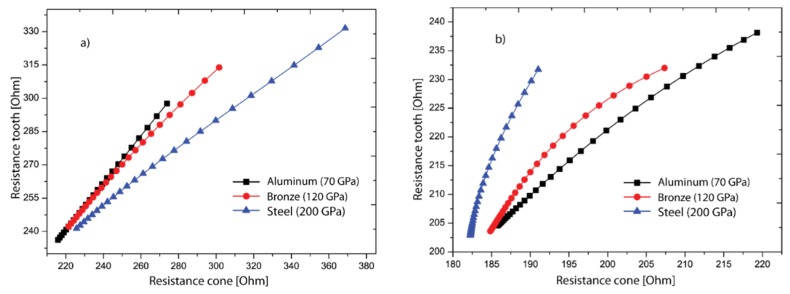
Relation between electrical resistance signals obtained with cone and premolar as coupling interfaces: (**a**) 9.3–9.7 kHz. (**b**) 11.1–11.5 kHz.

Besides, the electrical resistances were correlated with the purpose of verifying the relation between both coupling interfaces as illustrated in Figure 9a (9.3–9.7 kHz) and Figure 9b (11.1–11.5 kHz). As the results evidenced, those relations change structurally in both cases which allows to detect each elastic variation in the support. This could be considered another way of detection if both interfaces belong to the same structure. For example, in a real context, teeth are structures with different geometric and mechanical properties and these are embedded in bone. Correlation between measurements may be applied as a monitoring indicator. However, for future applications, all efforts should be focused in the description of the physics of each problem with the aim to understand the effects of structural variations over these relations.

## 4. Conclusions

The mechanical design and assessment of a piezo-actuated sensor for structural monitoring has been described, developed and experimentally tested. The electromechanical impedance technique was applied and its effects were investigated in order to detect elastic variations imposed in the support of the piezo-device. Experiments showed that when the elastic modulus was modified, the electrical resistance consistently varied in intervals 9.3–9.7 kHz and 11.1–11.5 kHz. This permitted the elastic variations to be quantified with an index based on RMSD. Two types of coupling interfaces (cone and human tooth) were tested to evidence the repeatability of the analysis presented in this study. Considering both coupling interfaces (joining part between support and piezo-device), the correlation among its electrical resistances were shown to be effective and sensitive to the elastic variations. Therefore, these changes were identified with the correlations. To find a potential applicability, it is necessary to perform tests in bio-structures in which can be monitored the reduction or the increment of its global elasticity. We see an opportunity to apply this methodology on teeth since these are connected directly with bone. Bone is a sensitive structure in which metabolic changes are reflected in its structures. However, it is important to consider that this paper provided a proof-of-concept that should be validated before a real bio-application.
